# Celebrating and supporting early career researchers within underrepresented groups in materials science

**DOI:** 10.1038/s41467-024-48495-8

**Published:** 2024-05-13

## Abstract

*Nature Communications* has been striving to support Early Career Researchers (ECRs) through different pilot schemes including the peer review mentoring programs and co-review mentoring initiative. The 2^nd^ Rising Stars workshop, held at the Henry Royce Institute on the 9^th^ of February, 2024, aims to celebrate and support rising stars within underrepresented groups in Science, Technology, Engineering and Mathematics (STEM) subjects and this greatly aligns with the aspirations in our journal. In this conversation, the experiences and advice shared by representatives from various disciplines in the workshop are translated to a wider audience in *Nature Communications*. Dr Alex Ramadan (Lecturer at the University of Sheffield), Dr Lucy Whalley (Assistant Professor at Northumbria University), Dr Maddison Coke (Senior Experimental Officer at the University of Manchester), and Dr Yi Liu (Lecturer at Loughborough University) discuss the opportunities and challenges they face towards their career with work-life balance, family and caring responsibility, and diversity and inclusion in their workplace, and share their experiences on how mentorship supports their personal and professional growth.

**Figure:**
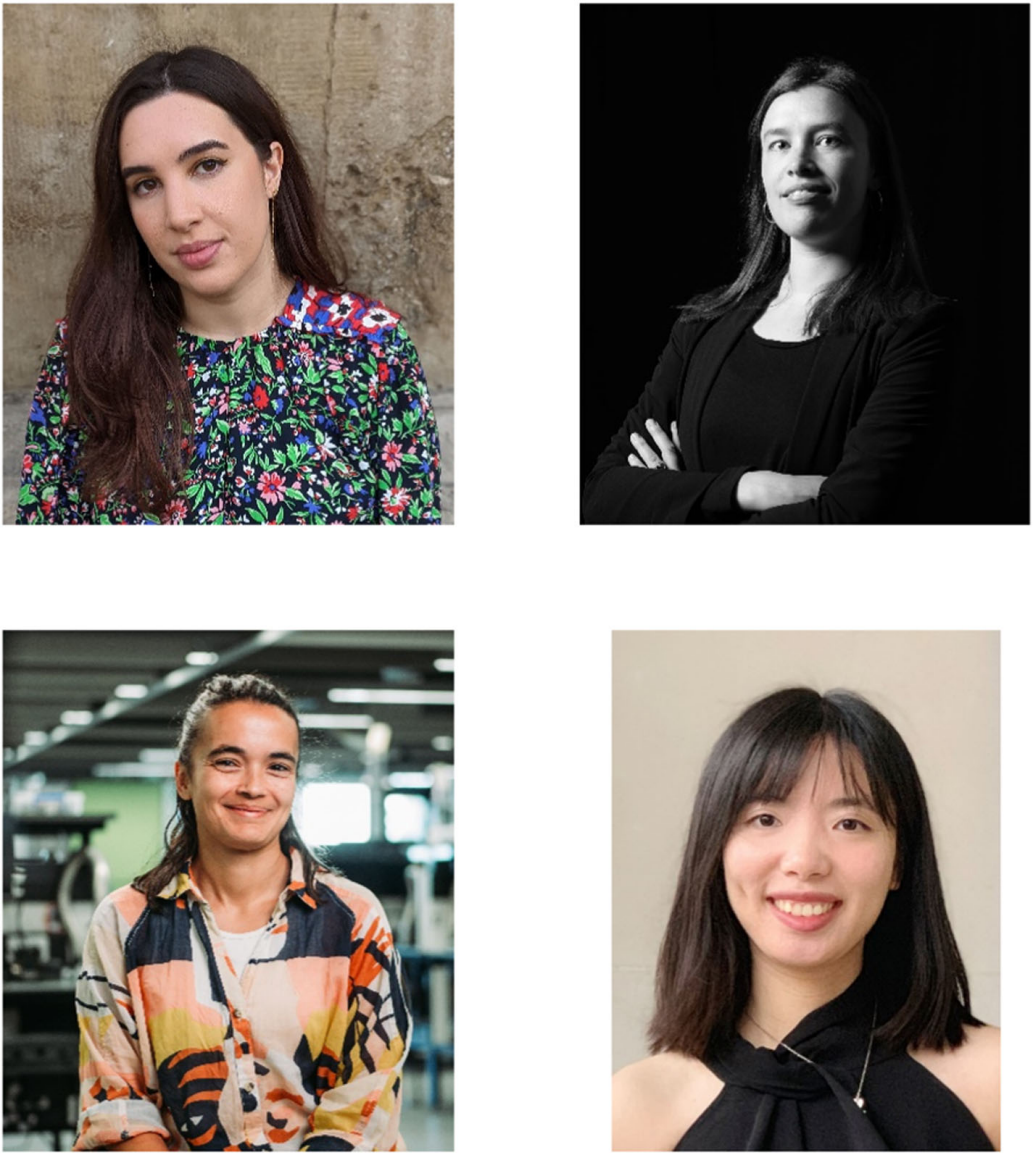
Top left: Alex Ramadan, Lecturer in Energy Materials at the University of Sheffield. Top right: Lucy Whalley, Assistant Professor of Physics at Northumbria University. Bottom left: Maddison Coke, Senior Experimental Officer at the Photon Science Institute at the University of Manchester. Bottom right: Yi Liu, Lecturer in Polymer Processing at Loughborough University. Robbie Oliver; Mark Slater; Alin-Bogdan Niculescu; Yi Liu.

1. Please introduce yourself. What has been the favourite part of your work so far?

**Alex Ramadan:** I’m a Lecturer in the Department of Physics and Astronomy at the University of Sheffield. I’m an experimental scientist working on advancing the scientific understanding and development of emerging semiconductors for implementation in optoelectronics, with a strong focus on photovoltaic devices. Alongside my research, I’m keen to improve research culture and equality, diversity, and inclusion in physical sciences. The favourite part of my work has always been the opportunity to meet new people, and to work with and learn from them. This year, I started supervising my first PhD students and working with them has been so enjoyable. I feel very lucky to get the opportunity to train the next generation scientists.

**Lucy Whalley:** I’m an Assistant Professor of Physics at Northumbria University. I use computational techniques to model materials for energy generation and storage. I also work to promote open software practices and volunteer as an Associate Editor at the Journal of Open Source Software. A highlight from the last few years has been undergraduate teaching, as it brings a nice contrast against research. I find that after a period of thinking deeply about my own research, stepping back and refreshing on the fundamentals for teaching is a welcome change.

**Maddison Coke:** I am a Senior Experimental Officer at the University of Manchester. The central part of my research work is the use of a focused ion beam for localised doping and single ion implantation. This has significant implications for future quantum technologies and wide-ranging technologies spanning from photonics to superconductors. The favourite part of my role is the variation in running local projects as well as being part of the national and global projects. Although managing expectations and requests is challenging, I have had many interactions with leaders in the field, which I relish, and there are always new challenges to solve.

**Yi Liu:** I am a Lecturer at Loughborough University, working on the development of smart and sustainable materials and their manufacturing methods to address the pressing environmental challenges. The favourite part of my work has been the opportunity to lead collaborative research projects ranging from energy-efficient manufacturing to plastic recycling. The process of translating innovative ideas into tangible solutions has been incredibly rewarding. I enjoy engaging with students and peers in these endeavours, which not only fuels my passion for research but also reinforces my commitment to contributing to a sustainable future.

2. What has been the biggest challenge in establishing your career as an early career researcher?

**Alex Ramadan:** Landing a faculty position is the most challenging task I’ve faced. It felt like an impossible task, and it can be very difficult and demotivating, given the time spent on applications with only minimal feedback received. Having supportive mentors and colleagues, who offered honest advice and encouragement, has helped me to get through this challenge. In this process, I learnt that people are truly helpful and not to feel like it’s an imposition to ask people for their feedback.

**Lucy Whalley:** The biggest challenge has been balancing my work with having a baby, and now, a young child. As is the case for many parents in academia, my pregnancy and early years as a mother almost perfectly aligned with a work-intensive time trying to establish myself as an independent researcher. Sometimes, maintaining both roles felt almost impossible, and I struggled with stress and the lack of sleep. I’m pleased to say that time has passed now, as I’ve become comfortable in both of my new roles.

**Maddison Coke:** Taking risks. I am quite a risk-averse person; when it came to the end of my PhD, I struggled with the uncertainty and ultimately went for an Experimental Officer role, which was a permanent role and provided a sense of security. After three years, I felt that I needed more challenge and growth, and so I applied for my current job, which was on a fixed-term contract. I have felt much more fulfilled in this role and am glad that I took the risk and pushed myself beyond limit.

**Yi Liu:** I think the most challenging part has been time management. Maintaining a good balance among research, teaching, grant writing, administration and outreach works has been a formidable task. This is further complicated by the commitment to my private life, especially as a new parent and to attend to the unexpected illness of my baby. These have tested my resilience and stress management skills, compelling me to adapt quickly and re-prioritise important tasks to maintain the quality of my work and at the same time to spend precious moments with my family.

3. What are the resources available to you as an early career researcher? What kind of resources you wish you had to support you?

**Alex Ramadan:** Every institution I have worked at has offered training courses and a forum to support ECRs, in addition to the mentorship from colleagues. It can often be difficult to get all the information you need from one place; and therefore, sometimes an informal conversation with someone can prove far more valuable than generic resources available online.

**Lucy Whalley:** The main resource I have is the network of researchers around me. I worked in a very friendly and supportive research group during my PhD years. Recently, I was lucky to be one of five ECRs hired in the field of photovoltaics at Northumbria. This has created an informal peer cohort to support me through my transition to an academic position. To better accommodate time for research and teaching, I wish to have relief from some mundane administrative tasks, and to have a better guidance and policy that supports part-time working in academia.

**Maddison Coke:** Having honest conversations with my supervisor was one of the best resources for me. Throughout my PhD, I learnt to respect for what the seniors say with a balanced approach instead of just listening and taking everything as fact. I am lucky to have had people who champion me and my growth, with leaders to endorse and support me. This has helped with my career progression and the visibility for what I do.

**Yi Liu:** I am fortunate to be part of a highly supportive academic community at Loughborough University, where I collaborate with dedicated colleagues across campus. Additionally, I have the privilege of working alongside talented PhD students whose enthusiasm and innovative thinking greatly enhance our projects. I would appreciate a more robust funding mechanism to support PhDs, especially for international students with great potentials.

4. Do you have mentorship experiences supporting your personal and professional growth? What is your experience so far?

**Alex Ramadan:** I’ve had brilliant mentors throughout my academic journey, with one formal mentor and many unofficial mentors at different career stages. I have found them through my network, either by directly approaching them or being connected through someone I know. Having mentors at different career stages is valuable to me, be they well-established professors, people at my career stage or just above mine. I have some incredible friends and collaborators whose mentorship is just as valuable to me as that from world-renowned professors.

**Maddison Coke:** I have had official and unofficial mentorship, ranging from professional development to public engagement leadership, and have managed to get something out of every experience. Through setting a goal, being open-minded, and being ready to listen and talk, I gained a much better understanding of the university as a whole organisation from being mentored by a director of an institution hub, and also insights into strategic planning from my outreach leadership mentor, which are equally important to me.

**Yi Liu:** I have been receiving guidance from my mentor, Professor Eileen Yu, who generously share her experiences and insights in helping me to navigate challenges in academia and has inspired me to pass on the knowledge and support that I’ve received. This reciprocal relationship has been a cornerstone of my personal and professional development, which has enlightened me to place importance on empathy, guidance, and the sharing of experiences to the community.

5. Do you find your working environment diverse and inclusive enough? Any suggestion for improvement?

**Alex Ramadan:** I want my research group to be diverse, inclusive, and supportive and to be an environment that members can be their authentic selves. However, in my opinion, there is a lot of work to be done in the Physics discipline before I would describe it as diverse and inclusive. I think improvements can be made by everyone taking an active role to embed the principles of equality, diversity, and inclusion across everything, and to bear in mind that we can’t treat improving our working environment as a separate activity to the research, teaching and community activities. For example, to develop teaching materials which are accessible to all students at the point of developing a course rather than in response to complaints; and to improve diversity in research by taking a proactive approach towards supporting ECRs through invited talks, peer review opportunities and awards. The Rising Stars events were born out of our desire to make materials science more diverse and inclusive by providing career development, support, and a network for ECRs from marginalised communities.

**Lucy Whalley:** Academia is not an accurate reflection of the wider society, particularly the massive underrepresentation of minority groups in scientific research. To start addressing this, we can initiate frank conversations and dedicate more resources towards supporting underrepresented groups. I’d also like to see our professional organisations campaign for less travel restrictions, as many of my colleagues from the Global South cannot attend conferences in Europe because of visa delays or denials.

**Maddison Coke:** I have always gravitated towards groups and people where diversity is a strength. Being a woman in STEM, bisexual, biracial, and dyslexic, I feel comfortable being surrounded by people to bring my whole self to work. I have found that universities, on the whole, have been positive in this respect. That said, there are still discrepancies between the work force presented in science compared with the national demographic. I believe having role models at various levels and with different backgrounds can help improve diversity, equality and inclusion in the scientific community; but, at the same time, we need to be cautious about how those role models are selected, and not to heavily rely on few selected individuals who might only represent a small group of people.

**Yi Liu:** My working environment has been striving towards promoting diversity and inclusivity, thanks to the dedicated efforts of the Equality, Diversity, and Inclusion committee in our school. I’ve had the opportunity to organise activities to enhance our workspace for everyone; particularly, we focus on creating interactive programs to educate and foster a deeper understanding and appreciation of diversity. We also aim to improve mechanisms on receiving feedback, allowing staff and students to share their experiences and provide suggestions for our environment to be more welcoming and supportive. We hope to come up with flexible policies to better support work-life balance and caregiving responsibilities.

6. How do you make a balance between career-minded and family-oriented, especially if you have a childcare responsibility?

**Lucy Whalley:** I have a very supportive partner who is happy and able to cover childcare whilst I go to conferences or meet deadlines. I choose to work part-time (0.8 full-time equivalent) and use Friday as a “mop-up” day. Earlier in the week, I try to protect a block of time for undisturbed reading, writing and research, by hiding in a café to free my mind from admin tasks or the squeals of a four-year-old. With these, I can be more present at home in the weekday evenings and during weekends.

**Maddison Coke:** I don’t have any children, but my family – my wife and dog, who are very important to me. I have been able to be more present and focused during my home times by balancing and drawing delicate boundaries. I rarely bring work home. I send the odd email, but I don’t sit at a desk and work. I realised I would never complete “science”. This realisation has led me to use my time better, embrace scheduling, and enjoy a happier family life.

**Yi Liu:** I’m fortunate to have considerable support from my partner and parents, which significantly alleviates the pressure on caring responsibilities. Having colleagues at a similar career stage, with their children at the similar age, has been invaluable to me. Not only they provide practical help but also advice to navigate both my professional and personal life, supporting me to find a balance between advancing my career and ensuring to keep my family at the top priority.

7. What would you suggest for young researchers considering a career in academia? Anything else you would like to share?

**Alex Ramadan:** I really enjoy what I do but working in academia can be difficult and it can be very easy to neglect your mental health, especially when falling into patterns of overworking and comparing yourself to others. My advice would be to build a strong group of friends and colleagues, who can support you and offer you kind but honest advice when you need it, and who you can share your frustrations and wins with. I’ve found it important to have things in my life outside of academia which bring me joy and I always try and make sure I fully celebrate the wins when they happen. I’d also encourage ECRs to make good use of their networks to find people who have career paths they’d like to explore and ask them for advice and to share their journey.

**Lucy Whalley:** One suggestion is to recognise and record your strengths and gains. I find it fairly easy to focus on what I have *not* achieved, so I started to log one success each month. Another suggestion is to try and connect with people who understand both the professional and personal aspects of your life. I can talk openly and honestly with the people I have met through our Rising Stars workshops, and it helps to have those bonds. We aim to organise the next Rising Stars, hopefully in the coming January – if you would like to receive news about the next Rising Stars event, you can visit the Royce website for updates.

**Maddison Coke:** Explore your options and know why you want it. A career in research doesn’t have to look just one way. There are more diverse routes into and around academia now; knowing these routes and finding the best balance you want in your life is the most important. Knowing why you want to be an academic is important, as it’s not always going to go linearly in the right direction, but this will help you focus on key deliverables that are needed for you to achieve your goals.

**Yi Liu:** My advice is to embrace resilience and adaptability. It’s crucial to develop a diverse skill set that extends beyond your research niche. Networking is indispensable as it helps you to connect within and beyond your field. Actively seek mentorship for invaluable guidance and support. Embrace interdisciplinary opportunities as they open new doors and expand your perspective. More importantly, never underestimate the power of having conversations to engage with peers, mentors, and even those outside your immediate expertise. These interactions can spark new ideas, provide a different viewpoint, and offer practical advice to navigate and embrace your challenges.

*This interview was conducted by Dr Natalie Lok Kwan Li*.

